# Associations of the circulating levels of cytokines with risk of amyotrophic lateral sclerosis: a Mendelian randomization study

**DOI:** 10.1186/s12916-023-02736-7

**Published:** 2023-02-03

**Authors:** Bin Liu, Linshuoshuo Lyu, Wenkai Zhou, Jie Song, Ding Ye, Yingying Mao, Guo-Bo Chen, Xiaohui Sun

**Affiliations:** 1 Department of Epidemiology, Zhejiang Chinese Medical University School of Public Health, Hangzhou, 310053 China; 2 Center for General Practice Medicine, Department of General Practice Medicine, Clinical Research Institute, Zhejiang Provincial People’s Hospital, People’s Hospital of Hangzhou Medical College, Hangzhou, Zhejiang China; 3 Key Laboratory of Endocrine Gland Diseases of Zhejiang Province, Hangzhou, Zhejiang China

**Keywords:** Amyotrophic lateral sclerosis, Cytokine, Genome-wide association study, Mendelian randomization, Single nucleotide polymorphisms

## Abstract

**Background:**

Amyotrophic lateral sclerosis (ALS) is a neurodegenerative disorder that is accompanied by muscle weakness and muscle atrophy, typically resulting in death within 3–5 years from the disease occurrence. Though the cause of ALS remains unclear, increasing evidence has suggested that inflammation is involved in the pathogenesis of ALS. Thus, we performed two-sample Mendelian randomization (MR) analyses to estimate the associations of circulating levels of cytokines and growth factors with the risk of ALS.

**Methods:**

Genetic instrumental variables for circulating cytokines and growth factors were identified from a genome-wide association study (GWAS) of 8293 European participants. Summary statistics of ALS were obtained from a GWAS including 20,806 ALS cases and 59,804 controls of European ancestry. We used the inverse-variance weighted (IVW) method as the primary analysis. To test the robustness of our results, we further performed the simple-median method, weighted-median method, MR-Egger regression, and MR pleiotropy residual sum and outlier test. Finally, a reverse MR analysis was performed to assess the possibility of reverse causation between ALS and the cytokines that we identified.

**Results:**

After Bonferroni correction, genetically predicted circulating level of basic fibroblast growth factor (FGF-basic) was suggestively associated with a lower risk of ALS [odds ratio (OR): 0.74, 95% confidence interval (95% CI): 0.60–0.92, *P* = 0.007]. We also observed suggestive evidence that interferon gamma-induced protein 10 (IP-10) was associated with a 10% higher risk of ALS (OR: 1.10, 95% CI: 1.03–1.17, *P* = 0.005) in the primary study. The results of sensitivity analyses were consistent.

**Conclusions:**

Our systematic MR analyses provided suggestive evidence to support causal associations of circulating FGF-basic and IP-10 with the risk of ALS. More studies are warranted to explore how these cytokines may affect the development of ALS.

**Supplementary Information:**

The online version contains supplementary material available at 10.1186/s12916-023-02736-7.

## Background

Amyotrophic lateral sclerosis (ALS) is a neurodegenerative disorder accompanied by muscle weakness and muscle atrophy due to the degeneration of motor neurons in the brain and spinal cord, typically resulting in death within 3–5 years from the disease occurrence [1]. It is estimated that in Europe and the USA, ALS affects 3 to 5 per 100,000 people [2, 3]. Moreover, approximately 800,000 people were expected to die in the USA due to ALS [2, 3]. Since there is no cure for ALS, understanding the pathogenic factors may provide insights into slowing down the progression of this disease.

Emerging studies have shown that several risk factors, such as genetic predisposition, smoking, and exercise, might play essential roles in the development of ALS [4–6]. Recently, accumulating evidence suggested that systemic inflammation might be related to the pathogenesis of ALS. The altered circulating levels of lymphocytes and monocyte have been reported in ALS patients [7, 8]. For example, a meta-analysis of 25 studies reported that the serum levels of interleukin-6 (IL), IL-8, and tumor necrosis factor (TNF) receptor 1 were higher in ALS patients than those in the controls [9]. The evidence from another two individual case-control studies revealed that the serum level of basic fibroblast growth factor (FGF-basic) was higher in ALS patients, while TNF-related apoptosis-inducing ligand (TRAIL) was lower [10, 11]. However, the conclusions of the associations between some cytokines and ALS risk remained inconsistent. A case-control study observed a higher level of monocyte chemotactic protein-1 (MCP-1) in ALS cases than that in controls, while another study found no differences [12, 13]. Considering the debates on these cytokines and the bias existing in traditional observational study designs, assessing the potential causality of inflammatory factors on ALS was still needed [14].

Mendelian randomization (MR) analysis is a method that uses genetic variants as instrumental variables (IVs) for the environmental factors and determines their potential causal associations with outcomes [15]. Since genetic variants are presumed to inherit randomly, and alleles are not influenced by diseases, this method can minimize the influence of confounding factors and reverse causation bias [15]. Recently, a genome-wide association study (GWAS) meta-analysis evaluated the genetic basis for 41 cytokines, which provided a possibility to study their associations with ALS. Therefore, by using a two-sample MR design, we systematically assessed the potential causal relationships between circulating cytokines and the risk of ALS. Furthermore, a reverse MR analysis was performed to evaluate the effect of ALS on cytokines.

## Methods

### Study design

**Fig. 1:**
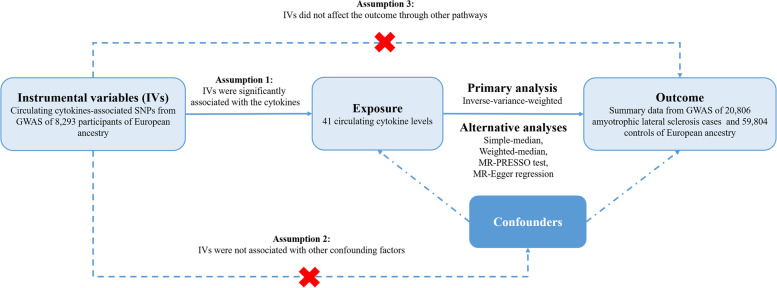
An overall design of the present study. *Abbreviations*: GWAS, genome-wide association study; MR, Mendelian randomization; MR-PRESSO, MR pleiotropy residual sum and outlier test; SNP, single nucleotide polymorphism

Figure 1 shows the overall design of our two-sample MR study. In order to obtain more credible results from the MR approach, our study tried to satisfy the following three assumptions of an MR study. First, the IVs were significantly associated with circulating levels of cytokines. Second, the IVs were not associated with other confounding factors. Finally, in addition to exposure factors, the IVs did not affect the outcome through other pathways [16]. Since we used publicly available GWAS summary statistics, no additional ethical approval was required.

### Data sources and instruments

#### Cytokines

The summary statistics and IVs for circulating levels of cytokines and growth factors were derived from a GWAS including 8293 European individuals [17]. Detailed information about this study is listed in Additional file 1: Table S1. To select the strong IVs, we first chose all single nucleotide polymorphisms (SNPs) that strongly and independently (*r*^2^ < 0.001 and distance = 250 kb) predicted cytokines at genome-wide significance (*P* < 5 × 10^−8^). Since the majority of the cytokines had no or limited (< 3) SNPs at *P* < 5 × 10^−8^, we further used a relaxed significance threshold (*P* < 5 × 10^−6^) to select IVs (Additional file 1: Tables S2 and S3). After clumping (*r*^2^ = 0.001, distance = 250 kb), a total of 625 SNPs associated with 41 cytokines were identified. To reduce potential pleiotropy across the SNPs, we excluded 44 SNPs that showed associations of *P* < 5 × 10^−6^ with more than one circulating cytokine. After removing 28 SNPs that were unavailable in the ALS dataset, a total of 553 SNPs associated with 41 cytokines were finally used as IVs in the present study. The number of the SNPs we used in the present study is listed in Additional file 1: Table S2.

#### Amyotrophic lateral sclerosis

The GWAS summary data of ALS were obtained from a meta-analysis of GWAS including 20,806 ALS cases and 59,804 controls of European ancestry [18]. Briefly, ALS patients were diagnosed by neurologists based on the EI Escorial criteria, which has been described in detail elsewhere [18]. A total of 10 genome-wide significant (*P* < 5 × 10^−8^) SNPs were identified for ALS. After clumping (*r*^2^ = 0.001, distance = 250 kb), 8 SNPs were retained as independent IVs for ALS. By using these SNPs, the reverse MR analysis was performed to investigate the effect of genetic predisposition to ALS on cytokine levels.

#### Statistical analyses

After extracting the information on cytokine-associated SNPs, such as the effect alleles and their corresponding β values, the genetic variance for each cytokine was estimated using the formula previously described [19]. In addition, we calculated the *F*-statistic to evaluate the strength of the IVs [20]. The *F*-statistics of IVs used in the present study ranged from 11 to 789, suggesting the sufficient strength of IVs [21]. Finally, we used an online web tool (https://sb452.shinyapps.io/power/) to calculate the statistical power of each cytokine [22]. The details of the variances explained, *F*-statistics, and power of the IVs for each cytokine are listed in Additional file 1: Tables S2 and S3.

We used the inverse-variance weighted (IVW) method as the primary analysis to evaluate the associations of circulating levels of cytokines with the risk of ALS by combining the *β* values and the standard errors of the causal estimate from them [23]. To assess the robustness of primary analyses, we applied several sensitivity analyses. First, we used the simple-median method and weighted-median method to estimate the potential cause effects when IVs went against standard assumptions [24]. Furthermore, MR-Egger regression was performed to assess the presence of directional pleiotropy, in which *P*-values for intercept < 0.05 were considered statistically significant [25]. In addition, we used the MR pleiotropy residual sum and outlier (MR-PRESSO) test to identify the possible pleiotropic outliers and reassessed the causal effect estimates after removing outliers [26]. Finally, we searched for secondary phenotypes associated with IVs by using the GWAS Catalog (http://www.ebi.ac.uk/gwas, last accessed on June 27, 2022) and reran the MR analyses after removing the SNPs of possible pleiotropy as documented.

We corrected for multiple comparisons with the Bonferroni approach and set statistical significance at a *P*-value < 1.22 × 10^−3^ (0.05/41) based on the number of cytokines. If a *P*-value was between 1.22 × 10^−3^ and 0.05, we considered suggestive evidence for a potential causal association [27]. All MR analyses were performed in the R software (v 3.6.4) using “TwoSampleMR,” “MendelianRandomization,” and “MRPRESSO” packages [26, 28, 29].

## Results

Firstly, when we selected the SNPs with *P* < 5 × 10^−8^ and clumped at linkage disequilibrium (LD) *r*^2^ = 0.001, only 28 cytokines included more than one SNP as IVs (Additional file 1: Table S2). In addition, only 11 cytokines included more than 3 SNPs, and the genetic variants ranged from 0.2 to 36%. Moreover, there were no cytokines that satisfied the power > 50% to obtain a significant association with ALS. Considering the low genetic variance as well as the limited number of SNPs and low powers, we performed MR analyses by liberalizing the threshold of *P* value to 5 × 10^−6^. By using such criteria (*r*^2^ < 0.001, *P* < 5 × 10^−6^), a total of 625 SNPs associated with 41 cytokines were identified. The *F*-statistics of IVs were all > 10, suggesting the robustness of IVs. Among these 41 cytokines, 11 of them were with > 50% power, and 3 with > 80% power to detect significant associations. More details can be found in Additional file 1: Table S3.

**Fig. 2:**
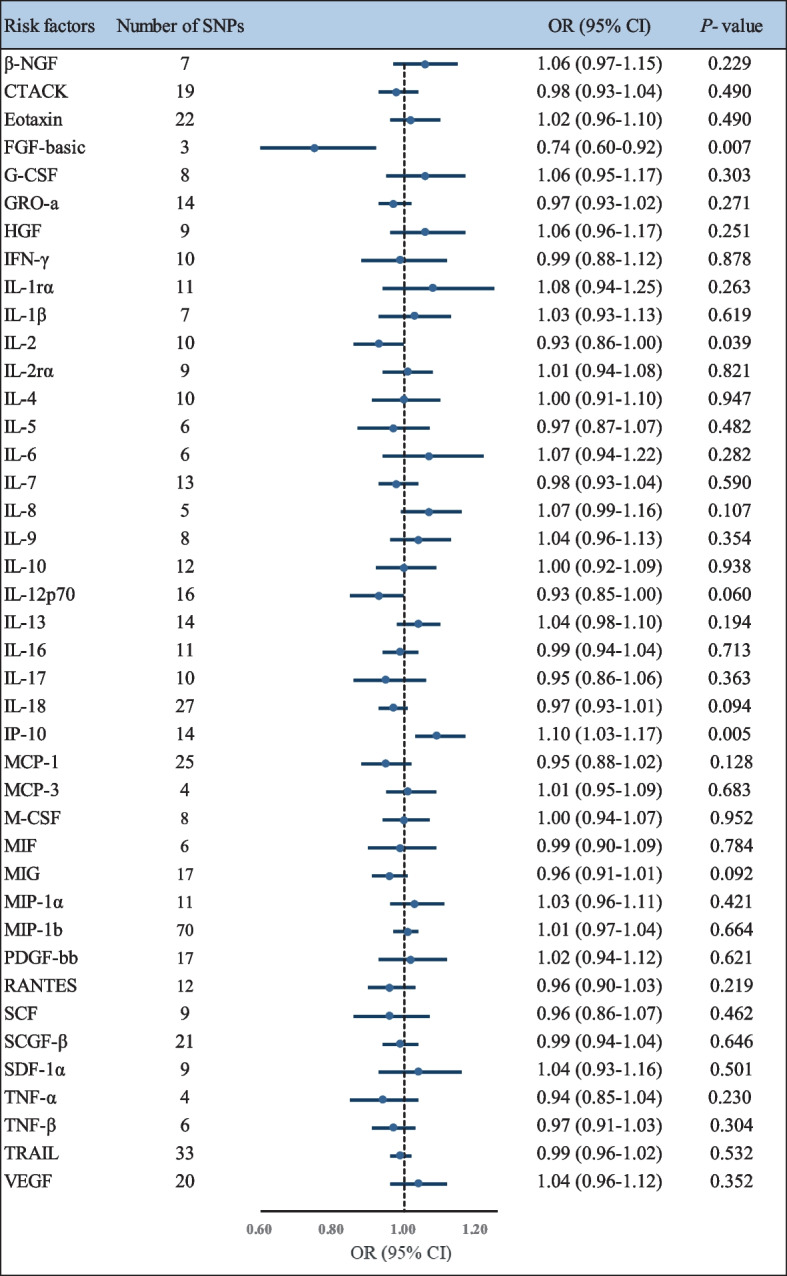
Forest plot of the Mendelian randomization analyses for the associations between circulating cytokines and risk of amyotrophic lateral sclerosis. *Abbreviations*: β-NGF, beta nerve growth factor; CI, confidence interval; CTACK, cutaneous T-cell attracting (CCL27); FGF-basic, basic fibroblast growth factor; G-CSF, granulocyte colony-stimulating factor; GRO-a, growth regulated oncogene-α (CXCL1); HGF, hepatocyte growth factor; IFN-γ, interferon-gamma; IL-1rα, interleukin-1 receptor antagonist; IL-1β, interleukin-1-beta; IL-2, interleukin-2; IL-2rα, interleukin-2 receptor, alpha subunit; IL-4, interleukin-4; IL-5, interleukin-5; IL-6, interleukin-6; IL-7, interleukin-7; IL-8, interleukin-8; IL-9, interleukin-9; IL-10, interleukin-10; IL-12p70, interleukin-12p70; IL-13, interleukin-13; IL-16, interleukin-16; IL-17, interleukin-17; IL-18, interleukin-18;IP-10, interferon gamma-induced protein 10 (CXCL10); MCP-1, monocyte chemotactic protein-1 (CCL2); MCP-3, monocyte specific chemokine 3 (CCL7); M-CSF, macrophage colony-stimulating factor; MIF, macrophage migration inhibitory factor; MIG, monokine induced by interferon-gamma; MIP-1α, macrophage inflammatory protein-1α (CCL3); MIP-1b, macrophage inflammatory protein-1β; OR, odds ratio; PDGF-bb, platelet derived growth factor BB; RANTES, regulated on activation, normal T-cell expressed and secreted (CCL5); SCF, stem cell factor; SCGF-β, stem cell growth factor beta; SDF-1α, stromal cell-derived factor-1 alpha; SNP, single nucleotide polymorphism; TNF-α, tumor necrosis factor-alpha; TNF-β, tumor necrosis factor-beta; TRAIL, TNF-related apoptosis inducing ligand; VEGF, vascular endothelial growth factor

The results from the IVW method of the relationships between 41 cytokines and ALS are presented in Fig. 2. After the Bonferroni correction, only three cytokines (FGF-basic, interferon gamma-induced protein 10 [IP-10], and IL-2) showed suggestive associations with ALS risk. Summary data for the associations of genetic variants are presented in Additional file 1: Table S4. Genetically determined higher circulating levels of FGF-basic showed a suggestive inverse association with the risk of ALS [odds ratio (OR): 0.74, 95% confidence interval (CI): 0.60–0.92, *P* = 0.007]. The results of the sensitivity analyses revealed similar but not statistically significant trends (OR: 0.75, 95% CI: 0.54–1.05, *P* = 0.098 by simple median method, OR: 0.75, 95% CI: 0.55–1.02, *P* = 0.063 by weighted median method). MR-Egger regression analyses did not detect a potential directional pleiotropy across the SNPs (intercept *P*-value = 0.639) (Additional file 1: Table S5). We further scanned the IVs in the GWAS Catalog and did not find any of the SNPs have been documented pleiotropy.

In addition, our results showed a suggestive adverse effect of the circulating level of IP-10 on ALS risk by the IVW method (OR: 1.10, 95% CI: 1.03–1.17, *P* = 0.005) (Additional file 1: Table S5). The results from the simple median method (OR: 1.11, 95% CI: 1.02–1.21, *P* = 0.020) and the weighted median method were consistent (OR: 1.10, 95% CI: 1.01–1.20, *P* = 0.029). The MR-PRESSO test also yielded a similar result (OR: 1.10, 95% CI: 1.04–1.16, *P* = 0.005). Furthermore, there was no evidence of pleiotropic effects as assessed by the intercept from MR-Egger regression (intercept *P*-value = 0.491). We found no SNPs associated with other traits when we searched the GWAS catalog.

We also noticed a suggestive association between genetically determined higher circulating IL-2 with a 7% lower risk of ALS (OR: 0.93, 95% CI: 0.86–1.00, *P* = 0.039). However, in sensitivity analyses, the association between IL-2 and ALS was not statistically significant (Additional file 1: Table S5). Moreover, we found that one SNP (rs1848347) was associated with other traits by searching the GWAS Catalog (Additional file 1: Table S6). After excluding this SNP, we observed no evidence of the association between genetically predicated IL-2 and ALS (OR: 0.94, 95% CI: 0.87–1.01, *P* = 0.092 by the IVW method) (Additional file 1: Table S7).

For reverse MR analyses, we did not observe statistically significant associations between ALS and IP-10 using the IVW method (OR: 0.92, 95% CI = 0.79–1.08, *P* = 0.302). However, we noticed a suggestive association between ALS risk and lower circulating levels of FGF-basic (OR: 0.87, 95% CI = 0.79–0.97, *P* = 0.015). The IVs we used and the results of other methods are listed in Additional file 1: Tables S8 and S9.

## Discussion

In the present study, we utilized the two-sample MR method to investigate the potential causal associations between circulating levels of 41 cytokines and the risk of ALS. We found suggestive evidence that the genetically predicted circulating levels of FGF-basic and IP-10 were associated with ALS.

FGF-basic was a member of the multifunctional protein family which can protect and develop the neural stem cells and the nervous system [30]. However, the epidemiological evidence for the relationship between FGF-basic and ALS was few, restricted by small sample sizes, and limited by using a case-control study design [10]. For example, a prior study including 15 ALS patients and 15 controls determined that the serum level of FGF-basic in ALS patients was higher than that in controls (11.17 ± 8.1 ng/L vs 1.26 ± 1.11 ng/L, *P* < 0.001) by using an immunoassay [10]. However, our MR analyses found a potential protective effect of circulating FGF-basic on ALS risk, indicating that the results of observational studies may need to be validated in further studies. The experimental studies using mouse models of ALS were also inconsistent in their conclusions. An animal study showed that a mouse model with FGF-basic treatment increased the proliferation of neural precursor cells and delayed the decrease in the density of S-IR boutons, supporting its protective effect on ALS risk [31]. It was reported that FGF-basic can reduce the activity of the Caspace3 gene and inhibit the active protein of Caspace3, thereby reducing apoptosis and protecting nerves [31]. However, Nadine et al. suggested that FGF-basic deficiency prolonged survival and improved motor in the ALS mouse model [32]. Notably, our reverse MR analysis showed a negative association between ALS and circulating FGF-basic levels. One possible explanation was that motoneuron loss in ALS patients may decrease the FGF-basic secreted [33, 34]. Given the inconsistency of the findings, more research is needed to determine the precise underlying mechanisms of FGF-basic in the development of ALS.

IP-10 is a type of cytokine that belongs to the CXC chemokine family and plays an important role in regulating cell growth and proliferation [35]. Only one observational study including 20 ALS cases and 20 controls previously reported no link between the serum level of IP-10 and ALS patients [12]. In contrast, we observed that a higher genetically predicted circulating IP-10 level was associated with a higher risk of ALS. The evidence from experimental studies supported our findings. It has been reported that the serum level of IP-10 in the SOD1 animal model of ALS increased with aging and is involved in disease progression [36]. Moreover, data from Caroline et al. showed that the IP10-directed chemotaxis mediated by the CXCR3 receptor could increase the migratory behavior of ALS lymphocytes when compared to healthy controls [37]. These data suggested that IP-10 may serve as a potential therapeutic target in ALS, but also need further studies to confirm the underlying biological mechanism. Furthermore, we noticed the serum level of IL-2 was associated with a lower risk of ALS in the primary analysis. However, there was no statistically significant association after excluding the SNPs with potentially pleiotropic effects. A meta-analysis including 25 studies also reported no association between serum levels of IL-2 and ALS [9]. Thus, the association between IL-2 and ALS may be a coincidental finding influenced by pleiotropic SNPs, or it could be a weak relationship due to the limitation of the IV used.

The associations between several circulating cytokines and ALS risk were evaluated by using the MR approach. Previously, Yuan et al. determined that a higher level of IL-1ra was significantly associated with a lower risk of ALS, while IL-2ra was not [38]. However, in the current study, we found no evidence of a link between IL-1ra or IL-2ra and ALS. Such differences could be driven by the different selections of IVs and GWAS summary data. Moreover, findings of another MR study showed no association between IL-6 level instrumented by one SNP and ALS risk, which is consistent with our results [39]. To the best of our knowledge, this is the first systematic and comprehensive MR study of the relationships between cytokines and ALS. Finally, we performed reverse MR analyses, which provided supportable evidence for our primary study.

However, several limitations of our study needed to be considered. First, since all the participants included in our study were restricted to European ancestry, our findings may not be generalizable to other races. Second, we used a relaxed significance threshold of *P* < 5 × 10^−6^ for the selection of IVs, and this might include false-positive variants and consequently bias. However, the *F*-statistics of IVs were all > 10, suggesting less likelihood of weak instrument bias. Moreover, we observed higher statistical power when compared to the restricted *P*-value cutoff (*P* < 5 × 10^−8^). Similarly, several other studies adopted the same significance threshold (*P* < 5 × 10^−6^) when they evaluated the associations between cytokines levels and Alzheimer’s disease, general cognitive function [40, 41]. Third, though we scanned the GWAS catalog to find potential secondary phenotypes of IVs, we cannot totally rule out the possibility of pleiotropy. Fourth, after the Bonferroni correction, no cytokine showed a statistically significant association with ALS risk, and only three of them (FGF-basic, IL-2, and IP-10) showed suggestive associations. With the exception of IL-2, all of these cytokines had statistical power of more than 80%; nonetheless, validation of these potential associations in larger cohorts and GWAS are needed. Moreover, it is possible that cytokines do not play a causal role in the development of ALS but still affect survival or disease progression [42–44]. However, our MR analysis did not address this association, and therefore, further studies should analyze if cytokines play a role in ALS aggressiveness.

## Conclusions

Our MR study supported potential causal associations of two circulating cytokines (FGF-basic and IP-10) with altered risk of ALS. Further studies are warranted to confirm these results, explore the underlying biological mechanisms, and assess whether they can serve as potential therapeutic targets.

## Supplementary Information

**Additional file 1: Table S1.** Details of the genome-wide association studies and datasets used in this study. **Table S2.** Details of the number of genetic instruments and *F*-statistic for each cytokine and growth factor. **Table S3.** Details of the genetic variance and statistical power for each cytokine and growth factor. **Table S4.** Characteristics of the genetic variants associated with circulating levels of FGF-basic, IP-10 and IL-2 in this study. **Table S5.** Effect estimates of the associations between circulating levels of 41 cytokines and risk of amyotrophic lateral sclerosis in MR analyses. **Table S6.** Details of the genetic variants with potential pleiotropy among instrumental variables of IL-2. **Table S7.** Effect estimates of the associations of circulating levels of IL-2 with risk of amyotrophic lateral sclerosis after excluding potential pleiotropic SNPs. **Table S8.** Characteristics of the genetic variants associated with amyotrophic lateral sclerosis. **Table S9.** Effect estimates of the associations of amyotrophic lateral sclerosis with risk of circulating levels of FGF-basic and IP-10.

## Data Availability

The data generated or analyzed during this study are available in this published article and its supplementary information files.

## Abbreviations

ALS: Amyotrophic lateral sclerosis
β-NGF: Beta nerve growth factor
CI: Confidence interval
CTACK: Cutaneous T-cell attracting (CCL27)
FGF-basic: Basic fibroblast growth factor
G-CSF: Granulocyte colony-stimulating factor
GRO-a: Growth regulated oncogene-α (CXCL1)
GWAS: Genome-wide association study
HGF: Hepatocyte growth factor
IFN-γ: Interferon-gamma
IL-1rα: Interleukin-1 receptor antagonist
IL-1β: Interleukin-1-beta
IL-2: Interleukin-2
IL-2rα: Interleukin-2 receptor, alpha subunit
IL-4: Interleukin-4
IL-5: Interleukin-5
IL-6: Interleukin-6
IL-7: Interleukin-7
IL-8: Interleukin-8
IL-9: Interleukin-9
IL-10: Interleukin-10
IL-12p70: Interleukin-12p70
IL-13: Interleukin-13
IL-16: Interleukin-16
IL-17: Interleukin-17
IL-18: Interleukin-18
IP-10: Interferon gamma-induced protein 10 (CXCL10)
IVs: Instrumental variables
IVW: Inverse-variance weighted
LD: Linkage disequilibrium
MCP-1: Monocyte chemotactic protein-1
MCP-3: Monocyte specific chemokine 3 (CCL7)
M-CSF: Macrophage colony-stimulating factor
MIF: Macrophage migration inhibitory factor
MIG: Monokine induced by interferon-gamma
MIP-1α: Macrophage inflammatory protein-1α (CCL3)
MIP-1b: Macrophage inflammatory protein-1β
MR: Mendelian randomization
MR-PRESSO: MR pleiotropy residual sum and outlier
OR: Odds ratio
PDGF-bb: Platelet-derived growth factor BB
RANTES: Regulated on activation, normal T-cell expressed and secreted (CCL5)
SCF: Stem cell factor
SCGF-β: Stem cell growth factor beta
SDF-1α: Stromal cell-derived factor-1 alpha
SNP: Single nucleotide polymorphism
TNF-α: Tumor necrosis factor-alpha
TNF-β: Tumor necrosis factor-beta
TRAIL: TNF-related apoptosis-inducing ligand
VEGF: Vascular endothelial growth factor
