# Supplementary material for: Associations of the circulating levels of cytokines with risk of amyotrophic lateral sclerosis: a Mendelian randomization study
Source: BMC Med. 2023 Feb 3;21:39. doi: 10.1186/s12916-023-02736-7 (PMC9898905; doi:10.1186/s12916-023-02736-7)
Supplement: Supplementary file 1 — Additional file 1: Table S1. Details of the genome-wide association studies and datasets used in this study. Table S2. Details of the number of genetic instruments and F-statistic for each cytokine and growth factor. Table S3. Details of the genetic variance and statistical power for each cytokine and growth factor. Table S4. Characteristics of the genetic variants associated with circulating levels of FGF-basic, IP-10 and IL-2 in this study. Table S5. Effect estimates of the associations between circulating levels of 41 cytokines and risk of amyotrophic lateral sclerosis in MR analyses. Table S6. Details of the genetic variants with potential pleiotropy among instrumental variables of IL-2. Table S7. Effect estimates of the associations of circulating levels of IL-2 with risk of amyotrophic lateral sclerosis after excluding potential pleiotropic SNPs. Table S8. Characteristics of the genetic variants associated with amyotrophic lateral sclerosis. Table S9. Effect estimates of the associations of amyotrophic lateral sclerosis with risk of circulating levels of FGF-basic and IP-10. [file 12916_2023_2736_MOESM1_ESM.docx]

Supplementary Materials for

Associations of the circulating levels of cytokines with risk of amyotrophic lateral sclerosis: a Mendelian randomization study

*Bin Liu, Linshuoshuo Lyu, Wenkai Zhou, Jie Song, Ding Ye, Yingying Mao, Guobo Chen, Xiaohui Sun*

**Table S1.** Details of the genome-wide association studies and datasets used in this study.

**Table S2.** Details of the number of genetic instruments and *F*-statistic for each cytokine and growth factor.

Abbreviations: No., number; SNP, single nucleotide polymorphism.

**Table S3.** Details of the genetic variance and statistical power for each cytokine and growth factor.

**Table S4.** Characteristics of the genetic variants associated with circulating levels of FGF-basic, IP-10 and IL-2 in this study.

Abbreviations: Chr, chromosome; FGF-basic, basic fibroblast growth factor; IL-2, interleukin-2; IP-10, interferon gamma-induced protein 10 (CXCL10); SE, standard error; SNP, single nucleotide polymorphism.

**Table S5.** Effect estimates of the associations between circulating levels of 41 cytokines and risk of amyotrophic lateral sclerosis in MR analyses.

Abbreviations: β-NGF, beta nerve growth factor; CI, confidence interval; CTACK, cutaneous T-cell attracting (CCL27); FGF-basic, basic fibroblast growth factor; G-CSF, granulocyte colony-stimulating factor; GRO-a, growth regulated oncogene-α (CXCL1); HGF, hepatocyte growth factor; IFN-γ, interferon-gamma; IL-1rα, interleukin-1 receptor antagonist; IL-1β, interleukin-1-beta; IL-2, interleukin-2; IL-2rα, interleukin-2 receptor, alpha subunit; IL-4, interleukin-4; IL-5, interleukin-5; IL-6, interleukin-6; IL-7, interleukin-7; IL-8, interleukin-8; IL-9, interleukin-9; IL-10, interleukin-10; IL-12p70, interleukin-12p70; IL-13, interleukin-13; IL-16, interleukin-16; IL-17, interleukin-17; IL-18, interleukin-18; IP-10, interferon gamma-induced protein 10 (CXCL10); MCP-1, monocyte chemotactic protein-1; MCP-3, monocyte specific chemokine 3 (CCL7); M-CSF, macrophage colony-stimulating factor; MIF, macrophage migration inhibitory factor; MIG, monokine induced by interferon-gamma; MIP-1α, macrophage inflammatory protein-1α (CCL3); MIP-1b, macrophage inflammatory protein-1β; MR, Mendelian randomization; MR-PRESSO,MR pleiotropy residual sum and outlier; OR, odds ratio; PDGF-bb, platelet derived growth factor BB; RANTES, regulated on activation normal T Cell expressed and secreted (CCL5); SCF, stem cell factor; SCGF-β, stem cell growth factor beta; SDF-1α, stromal cell-derived factor-1 alpha; SNP, single nucleotide polymorphism; TNF-α, tumor necrosis factor-alpha; TNF-β, tumor necrosis factor-beta; TRAIL, TNF-related apoptosis inducing ligand; VEGF, vascular endothelial growth factor. **P*-value of the intercept from MR-Egger regression analysis.

**Table S6.** Details of the genetic variants with potential pleiotropy among instrumental variables of IL-2.

*From the GWAS Catalog (http://www.ebi.ac.uk/gwas, last accessed on June 27th, 2022)

**Table S7.** Effect estimates of the associations of circulating levels of IL-2 with risk of amyotrophic lateral sclerosis after excluding potential pleiotropic SNPs.

Abbreviations: CI, confidence interval; IL-2, interleukin-2; MR, Mendelian randomization; MR-PRESSO test, MR Pleiotropy RESidual Sum and Outlier test; OR, odds ratio; SNP, single nucleotide polymorphism. **P*-value of the intercept from MR-Egger regression analysis.

**Table S8.** Characteristics of the genetic variants associated with amyotrophic lateral sclerosis.

Abbreviations: Chr, chromosome; SNP, single nucleotide polymorphism.

**Table S9.** Effect estimates of the associations of amyotrophic lateral sclerosis with risk of circulating levels of FGF-basic and IP-10.

Abbreviations: CI, confidence interval; FGF-basic, basic fibroblast growth factor; IP-10, interferon gamma-induced protein 10 (CXCL10); MR-PRESSO test, MR Pleiotropy RESidual Sum and Outlier test; OR, odds ratio; SNP, single nucleotide polymorphism. **P*-value of the intercept from MR-Egger regression analysis.

| **Table S1.** Details of the genome-wide association studies and datasets used in this study. | | | | |
| --- | --- | --- | --- | --- |
| Exposure or outcome | Sample size | Ancestry | Links for data | PMID |
| Circulating levels of 41 cytokines and growth factors | 8,293 participants | European ancestry | http://computationalmedicine.fi/data#  Cytokine_GWAS | 27989323 |
| Amyotrophic lateral sclerosis | 20,806 cases and  59,804 controls | European ancestry | http://als.umassmed.edu | 29566793 |

**Table S2.** Details of the number of genetic instruments and *F*-statistic for each cytokine and growth factor.

| Cytokines/ Growth factors | Abbreviations | *P* < 5 ×10^-8^ | |  | *P* < 5 ×10^-6^ | |
| --- | --- | --- | --- | --- | --- | --- |
|  |  | No. of SNPs | *F*-statistic (range) |  | No. of SNPs | *F*-statistic (range) |
| Beta nerve growth factor | β-NGF | 1 | 36.50 |  | 7 | 21.95(21.16-36.50) |
| Cutaneous T-cell attracting (CCL27) | CTACK | 4 | 37.80 (29.88-54.75) |  | 19 | 22.84 (20.83-37.48) |
| Eotaxin (CCL11) | Eotaxin | 8 | 33.38 (31.29-65.09) |  | 22 | 21.94 (20.81-55.38) |
| Basic fibroblast growth factor | FGF-basic | 0 | / |  | 3 | 21.65 (20.97-22.73) |
| Granulocyte colony-stimulating factor | G-CSF | 0 | / |  | 8 | 21.64 (20.36-22.48) |
| Growth regulated oncogene-α (CXCL1) | GRO-a | 1 | 30.19 |  | 14 | 22.02 (20.67-32.30) |
| Hepatocyte growth factor | HGF | 2 | 44.61 (31.97-57.25) |  | 9 | 22.59 (20.65-57.25) |
| Interferon-gamma | IFN-γ | 1 | 32.34 |  | 10 | 22.27 (20.89-23.32) |
| Interleukin-1 receptor antagonist | IL-1rα | 0 | / |  | 11 | 22.21 (20.94-26.51) |
| Interleukin-1-beta | IL-1β | 0 | / |  | 7 | 15.35 (13.61-31.57) |
| Interleukin-2 | IL-2 | 0 | / |  | 10 | 21.36 (20.90-22.98) |
| Interleukin-2 receptor, alpha subunit | IL-2rα | 2 | 41.34 (41.27-41.40) |  | 9 | 22.19 (20.81-82.28) |
| Interleukin-4 | IL-4 | 0 | / |  | 10 | 22.33 (20.93-25.56) |
| Interleukin-5 | IL-5 | 1 | 31.76 |  | 6 | 22.30 (21.78-24.87) |
| Interleukin-6 | IL-6 | 0 | / |  | 6 | 21.83 (21.11-23.35) |
| Interleukin-7 | IL-7 | 1 | 135.90 |  | 13 | 22.59 (20.59-97.13) |
| Interleukin-8 (CXCL8) | IL-8 | 0 | / |  | 5 | 22.94 (21.39-25.84) |
| Interleukin-9 | IL-9 | 0 | / |  | 8 | 21.81 (21.23-22.36) |
| Interleukin-10 | IL-10 | 3 | 35.57 (31.71-191.46) |  | 12 | 23.44 (21.20-129.96) |
| Interleukin-12p70 | IL-12p70 | 6 | 31.19 (28.56-52.51) |  | 16 | 21.78 (20.78-50.31) |
| Interleukin-13 | IL-13 | 2 | 31.70 (30.12-33.29) |  | 14 | 23.27 (21.10-31.96) |
| Interleukin-16 | IL-16 | 4 | 44.80 (29.63-53.34) |  | 11 | 22.73 (20.21-53.34) |
| Interleukin-17 | IL-17 | 1 | 30.88 |  | 10 | 22.64 (20.41-29.76) |
| Interleukin-18 | IL-18 | 10 | 35.87 (29.93-80.48) |  | 27 | 23.01 (21.10-37.60) |
| Interferon gamma-induced protein 10 (CXCL10) | IP-10 | 2 | 31.58 (31.11-32.04) |  | 14 | 22.70 (21.00-27.70) |
| Monocyte chemotactic protein-1 (CCL2) | MCP-1 | 8 | 34.50 (30.76-60.87) |  | 25 | 22.08 (20.76-45.17) |
| Monocyte specific chemokine 3 (CCL7) | MCP-3 | 0 | / |  | 4 | 22.33 (21.34-25.68) |
| Macrophage colony-stimulating factor | M-CSF | 1 | 31.64 |  | 8 | 21.81 (20.76-24.65) |
| Macrophage migration inhibitory factor (glycosylation-inhibiting factor) | MIF | 1 | 39.05 |  | 6 | 24.81 (21.20-28.11) |
| Monokine induced by interferon-gamma (CXCL9) | MIG | 1 | 34.11 |  | 17 | 21.61 (18.97-28.86) |
| Macrophage inflammatory protein-1α (CCL3) | MIP-1α | 0 | / |  | 11 | 21.26 (20.98-22.67) |
| Macrophage inflammatory protein-1β (CCL4) | MIP-1b | 48 | 38.38 (29.98-789.15) |  | 70 | 28.72 (20.56-789.15) |
| Platelet derived growth factor BB | PDGF-bb | 5 | 31.18 (29.88-69.50) |  | 17 | 22.98 (21.00-55.60) |
| Regulated on activation, normal T Cell expressed and secreted (CCL5) | RANTES | 1 | 29.99 |  | 12 | 22.71 (20.87-27.14) |
| Stem cell factor | SCF | 1 | 30.56 |  | 9 | 22.27 (20.73-25.46) |
| Stem cell growth factor beta | SCGF-β | 7 | 42.23 (34.48-52.07) |  | 21 | 22.03 (20.61-52.07) |
| Stromal cell-derived factor-1 alpha (CXCL12) | SDF-1α | 0 | / |  | 9 | 19.42 (11.12-29.21) |
| Tumor necrosis factor-alpha | TNF-α | 0 | / |  | 4 | 22.60 (21.40-23.96) |
| Tumor necrosis factor-beta | TNF-β | 2 | 45.21 (39.62-50.80) |  | 6 | 22.59 (21.24-50.80) |
| TNF-related apoptosis inducing ligand | TRAIL | 15 | 46.93 (32.01-127.33) |  | 33 | 26.03 (19.86-352.41) |
| Vascular endothelial growth factor | VEGF | 10 | 35.75 (31.13-86.96) |  | 20 | 23.64 (20.66-71.20) |

Abbreviations: No., number; SNP, single nucleotide polymorphism.

**Table S3.** Details of the genetic variance and statistical power for each cytokine and growth factor.

| Cytokines/ Growth factors | Abbreviations | *P* < 5 ×10^-8^ | |  | *P* < 5 ×10^-6^ | |
| --- | --- | --- | --- | --- | --- | --- |
|  |  | Variance explained ^*^ | Odds ratio†  estimated for α<0.05 and power≥80% |  | Variance explained ^*^ | Odds ratio†  estimated for α<0.05 and power≥80% |
| Beta nerve growth factor | β-NGF | 0.011 | 1.239 |  | 0.043 | 1.115 |
| Cutaneous T-cell attracting (CCL27) | CTACK | 0.041 | 1.118 |  | 0.140 | 1.062 |
| Eotaxin (CCL11) | Eotaxin | 0.044 | 1.113 |  | 0.077 | 1.085 |
| Basic fibroblast growth factor | FGF-basic | / | / |  | 0.006 | 1.338 |
| Granulocyte colony-stimulating factor | G-CSF | / | / |  | 0.048 | 1.108 |
| Growth regulated oncogene-α (CXCL1) | GRO-a | 0.015 | 1.202 |  | 0.133 | 1.064 |
| Hepatocyte growth factor | HGF | 0.015 | 1.202 |  | 0.042 | 1.116 |
| Interferon-gamma | IFN-γ | 0.002 | 1.655 |  | 0.024 | 1.157 |
| Interleukin-1 receptor antagonist | IL-1rα | / | / |  | 0.049 | 1.107 |
| Interleukin-1-beta | IL-1β | / | / |  | 0.042 | 1.116 |
| Interleukin-2 | IL-2 | / | / |  | 0.063 | 1.094 |
| Interleukin-2 receptor, alpha subunit | IL-2rα | 0.012 | 1.229 |  | 0.082 | 1.082 |
| Interleukin-4 | IL-4 | / | / |  | 0.050 | 1.106 |
| Interleukin-5 | IL-5 | 0.010 | 1.253 |  | 0.046 | 1.111 |
| Interleukin-6 | IL-6 | / | / |  | 0.025 | 1.157 |
| Interleukin-7 | IL-7 | 0.044 | 1.113 |  | 0.148 | 1.060 |
| Interleukin-8 (CXCL8) | IL-8 | / | / |  | 0.056 | 1.100 |
| Interleukin-9 | IL-9 | / | / |  | 0.058 | 1.098 |
| Interleukin-10 | IL-10 | 0.040 | 1.119 |  | 0.054 | 1.102 |
| Interleukin-12p70 | IL-12p70 | 0.030 | 1.139 |  | 0.062 | 1.094 |
| Interleukin-13 | IL-13 | 0.015 | 1.202 |  | 0.124 | 1.066 |
| Interleukin-16 | IL-16 | 0.066 | 1.092 |  | 0.141 | 1.062 |
| Interleukin-17 | IL-17 | 0.004 | 1.422 |  | 0.036 | 1.126 |
| Interleukin-18 | IL-18 | 0.114 | 1.069 |  | 0.239 | 1.047 |
| Interferon gamma-induced protein 10 (CXCL10) | IP-10 | 0.019 | 1.178 |  | 0.096 | 1.075 |
| Monocyte chemotactic protein-1 (CCL2) | MCP-1 | 0.034 | 1.130 |  | 0.089 | 1.078 |
| Monocyte specific chemokine 3 (CCL7) | MCP-3 | / | / |  | 0.084 | 1.081 |
| Macrophage colony-stimulating factor | M-CSF | 0.016 | 1.195 |  | 0.127 | 1.065 |
| Macrophage migration inhibitory factor (glycosylation-inhibiting factor) | MIF | 0.011 | 1.239 |  | 0.050 | 1.106 |
| Monokine induced by interferon-gamma (CXCL9) | MIG | 0.008 | 1.286 |  | 0.175 | 1.055 |
| Macrophage inflammatory protein-1α (CCL3) | MIP-1α | / | / |  | 0.068 | 1.090 |
| Macrophage inflammatory protein-1β (CCL4) | MIP-1b | 0.355 | 1.039 |  | 0.394 | 1.037 |
| Platelet derived growth factor BB | PDGF-bb | 0.024 | 1.157 |  | 0.054 | 1.102 |
| Regulated on activation, normal T Cell expressed and secreted (CCL5) | RANTES | 0.004 | 1.422 |  | 0.074 | 1.087 |
| Stem cell factor | SCF | 0.004 | 1.422 |  | 0.036 | 1.126 |
| Stem cell growth factor beta | SCGF-β | 0.072 | 1.088 |  | 0.156 | 1.059 |
| Stromal cell-derived factor-1 alpha (CXCL12) | SDF-1α | / | / |  | 0.027 | 1.147 |
| Tumor necrosis factor-alpha | TNF-α | / | / |  | 0.064 | 1.093 |
| Tumor necrosis factor-beta | TNF-β | 0.049 | 1.107 |  | 0.112 | 1.070 |
| TNF-related apoptosis inducing ligand | TRAIL | 0.112 | 1.070 |  | 0.230 | 1.048 |
| Vascular endothelial growth factor | VEGF | 0.063 | 1.094 |  | 0.082 | 1.082 |

*. β: effect size estimates of the SNPs of cytokines/growth factors levels.; MAF: minimum allele frequency. The variance of each cytokine was calculated by using an additive model under the assumption of no interaction between each SNPs. (Georgakis et al. Circulation, 2019, 139(2): 256-268.)

| † The odds ratio per 1SD elevate in circulating cytokines/ growth factors levels, for which there is power (1-β)≥80% to detect an existed association at α<0.05. |
| --- |

**Table S4.** Characteristics of instrumental variables used for circulating levels of FGF-basic, IP-10 and IL-2 in this study.

| Cytokines/ Growth factors | SNP | Chr | Position | Effect allele | Beta | SE | *P*-value |
| --- | --- | --- | --- | --- | --- | --- | --- |
| FGF-basic | rs2118824 | 10 | 92738106 | g | -0.075 | 0.016 | 4.55×10^-06^ |
| FGF-basic | rs4796109 | 17 | 34138908 | g | 0.127 | 0.027 | 1.47×10-^06^ |
| FGF-basic | rs2849358 | 18 | 71085131 | g | -0.090 | 0.019 | 3.31×10-^06^ |
| IP-10 | rs7645625 | 3 | 146574037 | g | 0.109 | 0.024 | 4.41×10-^06^ |
| IP-10 | rs4859940 | 4 | 75988363 | g | 0.125 | 0.026 | 1.55×10-^06^ |
| IP-10 | rs79750500 | 4 | 76296440 | g | -0.337 | 0.071 | 2.01×10-^06^ |
| IP-10 | rs188759467 | 5 | 73103247 | t | -0.450 | 0.096 | 3.22×10-^06^ |
| IP-10 | rs113183470 | 6 | 86123575 | t | 0.250 | 0.053 | 1.96×10-^06^ |
| IP-10 | rs9450313 | 6 | 86394137 | t | 0.270 | 0.052 | 1.94×10-^07^ |
| IP-10 | rs9359671 | 6 | 86644697 | g | -0.263 | 0.051 | 2.67×10-^07^ |
| IP-10 | rs113032464 | 6 | 86918632 | g | -0.298 | 0.057 | 1.39×10-^07^ |
| IP-10 | rs34383175 | 8 | 145584694 | c | 0.315 | 0.066 | 1.51×10-^06^ |
| IP-10 | rs72708794 | 9 | 11041407 | c | -0.130 | 0.028 | 3.69×10-^06^ |
| IP-10 | rs1951169 | 14 | 36965881 | g | -0.115 | 0.025 | 2.47×10-^06^ |
| IP-10 | rs71401909 | 15 | 87216956 | t | 0.287 | 0.062 | 2.91×10-^06^ |
| IP-10 | rs8112618 | 19 | 46413398 | g | -0.142 | 0.030 | 2.02×10-^06^ |
| IP-10 | rs397816 | 22 | 22728326 | c | -0.124 | 0.025 | 7.90×10^-07^ |
| IL-2 | rs2690020 | 1 | 15199644 | g | -0.113 | 0.025 | 4.61×10-^06^ |
| IL-2 | rs62124990 | 2 | 19238636 | g | 0.696 | 0.150 | 3.22×10-^06^ |
| IL-2 | rs80336398 | 3 | 64060934 | c | -0.400 | 0.086 | 2.82×10-^06^ |
| IL-2 | rs1995425 | 3 | 156661809 | c | 0.115 | 0.025 | 3.94×10-^06^ |
| IL-2 | rs1848347 | 4 | 55386878 | g | 0.162 | 0.035 | 3.93×10-^06^ |
| IL-2 | rs4634519 | 7 | 67192928 | g | 0.126 | 0.027 | 2.77×10-^06^ |
| IL-2 | rs1949803 | 7 | 120869625 | g | -0.118 | 0.025 | 1.61×10-^06^ |
| IL-2 | rs10903540 | 10 | 1717507 | g | 0.158 | 0.034 | 4.19×10-^06^ |
| IL-2 | rs12051139 | 16 | 86918674 | c | 0.113 | 0.025 | 4.76×10-^06^ |
| IL-2 | rs56254584 | 21 | 15273744 | g | -0.130 | 0.029 | 4.84×10^-06^ |

Abbreviations: Chr, chromosome; FGF-basic, basic fibroblast growth factor; IL-2, interleukin-2; IP-10, interferon gamma-induced protein 10 (CXCL10); SE, standard error; SNP, single nucleotide polymorphism.

**Table S5.** Effect estimates of the associations between circulating levels of 41 cytokines and risk of amyotrophic lateral sclerosis in MR analyses.

| Methods | Number of SNPs | OR | 95% CI | *P-*value |
| --- | --- | --- | --- | --- |
| **β-NGF** |  |  |  |  |
| Inverse-variance weighted | 7 | 1.06 | 0.97-1.15 | 0.229 |
| Weighted median | 7 | 1.07 | 0.95-1.21 | 0.236 |
| Simple median | 7 | 1.06 | 0.93-1.20 | 0.406 |
| MR-PRESSO test | 7 | 1.06 | 0.96-1.16 | 0.318 |
| MR-Egger | 7 | / | / | 0.224* |
| **CTACK** |  |  |  |  |
| Inverse-variance weighted | 19 | 0.98 | 0.93-1.04 | 0.490 |
| Weighted median | 19 | 0.99 | 0.92-1.07 | 0.864 |
| Simple median | 19 | 0.99 | 0.92-1.08 | 0.874 |
| MR-PRESSO test | 19 | 0.98 | 0.94-1.03 | 0.417 |
| MR-Egger | 19 | / | / | 0.863* |
| **Eotaxin** |  |  |  |  |
| Inverse-variance weighted | 22 | 1.02 | 0.96-1.10 | 0.490 |
| Weighted median | 22 | 0.98 | 0.88-1.09 | 0.725 |
| Simple median | 22 | 1.02 | 0.92-1.14 | 0.674 |
| MR-PRESSO test | 22 | 1.02 | 0.94-1.12 | 0.581 |
| MR-Egger | 22 | 0.97 | 0.69-1.36 | 0.749* |
| **FGF-basic** |  |  |  |  |
| Inverse-variance weighted | 3 | 0.74 | 0.60-0.92 | 0.007 |
| Weighted median | 3 | 0.75 | 0.55-1.02 | 0.063 |
| Simple median | 3 | 0.75 | 0.54-1.05 | 0.098 |
| MR-PRESSO test | 3 | / | / | / |
| MR-Egger | 3 | / | / | 0.639* |
| **G-CSF** |  |  |  |  |
| Inverse-variance weighted | 8 | 1.06 | 0.95-1.17 | 0.303 |
| Weighted median | 8 | 1.01 | 0.88-1.16 | 0.875 |
| Simple median | 8 | 1.05 | 0.91-1.20 | 0.535 |
| MR-PRESSO test | 8 | 1.06 | 0.99-1.13 | 0.150 |
| MR-Egger | 8 | / | / | 0.435* |
| **GRO-a** |  |  |  |  |
| Inverse-variance weighted | 14 | 0.97 | 0.93-1.02 | 0.271 |
| Weighted median | 14 | 0.99 | 0.93-1.06 | 0.797 |
| Simple median | 14 | 0.96 | 0.90-1.03 | 0.271 |
| MR-PRESSO test | 14 | 0.97 | 0.93-1.02 | 0.307 |
| MR-Egger | 14 | / | / | 0.224* |
| **HGF** |  |  |  |  |
| Inverse-variance weighted | 9 | 1.06 | 0.96-1.17 | 0.251 |
| Weighted median | 9 | 1.10 | 0.97-1.26 | 0.138 |
| Simple median | 9 | 1.06 | 0.91-1.22 | 0.465 |
| MR-PRESSO test | 9 | 1.06 | 0.93-1.19 | 0.368 |
| MR-Egger | 9 | / | / | 0.452* |
| **IFN-γ** |  |  |  |  |
| Inverse-variance weighted | 10 | 0.99 | 0.88-1.12 | 0.878 |
| Weighted median | 10 | 1.05 | 0.89-1.24 | 0.539 |
| Simple median | 10 | 1.03 | 0.87-1.21 | 0.767 |
| MR-PRESSO test | 10 | 0.99 | 0.87-1.11 | 0.868 |
| MR-Egger | 10 | / | / | 0.028* |
| **IL-1rα** |  |  |  |  |
| Inverse-variance weighted | 11 | 1.08 | 0.94-1.25 | 0.263 |
| Weighted median | 11 | 1.09 | 0.96-1.23 | 0.183 |
| Simple median | 11 | 1.04 | 0.91-1.20 | 0.549 |
| MR-PRESSO test | 10 | 1.05 | 0.93-1.19 | 0.472 |
| MR-Egger | 11 | / | / | 0.746* |
| **IL-1β** |  |  |  |  |
| Inverse-variance weighted | 7 | 1.03 | 0.93-1.13 | 0.619 |
| Weighted median | 7 | 1.00 | 0.87-1.15 | 0.983 |
| Simple median | 7 | 1.01 | 0.86-1.19 | 0.906 |
| MR-PRESSO test | 7 | 1.03 | 0.93-1.13 | 0.629 |
| MR-Egger | 7 | / | / | 0.371* |
| **IL-2** |  |  |  |  |
| Inverse-variance weighted | 10 | 0.93 | 0.86-1.00 | 0.039 |
| Weighted median | 10 | 0.93 | 0.83-1.04 | 0.213 |
| Simple median | 10 | 0.92 | 0.83-1.02 | 0.168 |
| MR-PRESSO test | 10 | 0.93 | 0.84-1.02 | 0.160 |
| MR-Egger | 10 | / | / | 0.999* |
| **IL2ra** |  |  |  |  |
| Inverse-variance weighted | 9 | 1.01 | 0.94-1.08 | 0.821 |
| Weighted median | 9 | 1.01 | 0.92-1.11 | 0.811 |
| Simple median | 9 | 0.97 | 0.87-1.08 | 0.533 |
| MR-PRESSO test | 9 | 1.01 | 0.94-1.08 | 0.827 |
| MR-Egger | 9 | / | / | 0.531* |
| **IL-4** |  |  |  |  |
| Inverse-variance weighted | 10 | 1.00 | 0.91-1.10 | 0.947 |
| Weighted median | 10 | 1.01 | 0.89-1.15 | 0.850 |
| Simple median | 10 | 0.96 | 0.83-1.10 | 0.522 |
| MR-PRESSO test | 10 | 1.00 | 0.93-1.08 | 0.935 |
| MR-Egger | 10 | / | / | 0.304* |
| **IL-5** |  |  |  |  |
| Inverse-variance weighted | 6 | 0.97 | 0.87-1.07 | 0.482 |
| Weighted median | 6 | 0.99 | 0.87-1.12 | 0.827 |
| Simple median | 6 | 0.99 | 0.87-1.12 | 0.880 |
| MR-PRESSO test | 6 | 0.97 | 0.90-1.04 | 0.370 |
| MR-Egger | 6 | / | / | 0.864* |
| **IL-6** |  |  |  |  |
| Inverse-variance weighted | 6 | 1.07 | 0.94-1.22 | 0.282 |
| Weighted median | 6 | 1.09 | 0.91-1.31 | 0.353 |
| Simple median | 6 | 1.13 | 0.94-1.36 | 0.179 |
| MR-PRESSO test | 6 | 1.07 | 0.90-1.29 | 0.477 |
| MR-Egger | 6 | / | / | 0.583* |
| **IL-7** |  |  |  |  |
| Inverse-variance weighted | 13 | 0.98 | 0.93-1.04 | 0.590 |
| Weighted median | 13 | 0.98 | 0.91-1.06 | 0.651 |
| Simple median | 13 | 0.97 | 0.90-1.06 | 0.529 |
| MR-PRESSO test | 13 | 0.98 | 0.93-1.05 | 0.633 |
| MR-Egger | 13 | / | / | 0.899* |
| **IL-8** |  |  |  |  |
| Inverse-variance weighted | 5 | 1.07 | 0.99-1.16 | 0.107 |
| Weighted median | 5 | 1.07 | 0.96-1.20 | 0.238 |
| Simple median | 5 | 1.00 | 0.87-1.16 | 0.964 |
| MR-PRESSO test | 5 | 1.07 | 0.96-1.19 | 0.277 |
| MR-Egger | 5 | / | / | 0.467* |
| **IL-9** |  |  |  |  |
| Inverse-variance weighted | 8 | 1.04 | 0.96-1.13 | 0.354 |
| Weighted median | 8 | 1.02 | 0.92-1.13 | 0.658 |
| Simple median | 8 | 1.03 | 0.93-1.14 | 0.522 |
| MR-PRESSO test | 8 | 1.04 | 0.97-1.11 | 0.315 |
| MR-Egger | 8 | / | / | 0.327* |
| **IL-10** |  |  |  |  |
| Inverse-variance weighted | 12 | 1.00 | 0.92-1.09 | 0.938 |
| Weighted median | 12 | 0.99 | 0.88-1.10 | 0.822 |
| Simple median | 12 | 0.95 | 0.84-1.07 | 0.401 |
| MR-PRESSO test | 12 | 1.00 | 0.91-1.10 | 0.947 |
| MR-Egger | 12 | / | / | 0.642* |
| **IL-12p70** |  |  |  |  |
| Inverse-variance weighted | 16 | 0.93 | 0.85-1.00 | 0.060 |
| Weighted median | 16 | 0.93 | 0.83-0.15 | 0.245 |
| Simple median | 16 | 0.93 | 0.83-1.04 | 0.215 |
| MR-PRESSO test | 16 | 0.93 | 0.85-1.00 | 0.078 |
| MR-Egger | 16 | / | / | 0.263* |
| **IL-13** |  |  |  |  |
| Inverse-variance weighted | 14 | 1.04 | 0.98-1.10 | 0.194 |
| Weighted median | 14 | 1.03 | 0.95-1.12 | 0.477 |
| Simple median | 14 | 1.02 | 0.94-1.11 | 0.614 |
| MR-PRESSO test | 14 | 1.00 | 0.91-1.10 | 0.947 |
| MR-Egger | 14 | / | / | 0.055* |
| **IL-16** |  |  |  |  |
| Inverse-variance weighted | 11 | 0.99 | 0.94-1.04 | 0.713 |
| Weighted median | 11 | 0.99 | 0.92-1.07 | 0.794 |
| Simple median | 11 | 0.98 | 0.91-1.06 | 0.624 |
| MR-PRESSO test | 11 | 0.99 | 0.93-1.05 | 0.747 |
| MR-Egger | 11 | / | / | 0.538* |
| **IL-17** |  |  |  |  |
| Inverse-variance weighted | 10 | 0.95 | 0.86-1.06 | 0.363 |
| Weighted median | 10 | 0.90 | 0.78-1.04 | 0.159 |
| Simple median | 10 | 1.02 | 0.87-1.20 | 0.813 |
| MR-PRESSO test | 10 | 0.95 | 0.87-1.05 | 0.334 |
| MR-Egger | 10 | / | / | 0.462* |
| **IL-18** |  |  |  |  |
| Inverse-variance weighted | 27 | 0.97 | 0.93-1.01 | 0.094 |
| Weighted median | 27 | 0.96 | 0.90-1.03 | 0.246 |
| Simple median | 27 | 0.98 | 0.92-1.05 | 0.622 |
| MR-PRESSO test | 27 | 0.97 | 0.92-1.01 | 0.173 |
| MR-Egger | 27 | / | / | 0.858* |
| **IP-10** |  |  |  |  |
| Inverse-variance weighted | 14 | 1.10 | 1.03-1.17 | 0.005 |
| Weighted median | 14 | 1.10 | 1.01-1.20 | 0.029 |
| Simple median | 14 | 1.11 | 1.02-1.21 | 0.020 |
| MR-PRESSO test | 14 | 1.10 | 1.04-1.16 | 0.005 |
| MR-Egger | 14 | / | / | 0.491* |
| **MCP-1** |  |  |  |  |
| Inverse-variance weighted | 25 | 0.95 | 0.88-1.02 | 0.128 |
| Weighted median | 25 | 0.93 | 0.84-1.03 | 0.179 |
| Simple median | 25 | 0.92 | 0.83-1.02 | 0.124 |
| MR-PRESSO test | 25 | 0.95 | 0.87-1.03 | 0.217 |
| MR-Egger | 25 | / | / | 0.191* |
| **MCP-3** |  |  |  |  |
| Inverse-variance weighted | 4 | 1.01 | 0.95-1.09 | 0.683 |
| Weighted median | 4 | 1.02 | 0.94-1.10 | 0.636 |
| Simple median | 4 | 1.00 | 0.93-1.09 | 0.958 |
| MR-PRESSO test | 4 | 1.01 | 0.98-1.05 | 0.430 |
| MR-Egger | 4 | / | / | 0.699* |
| **M-CSF** |  |  |  |  |
| Inverse-variance weighted | 8 | 1.00 | 0.94-1.07 | 0.952 |
| Weighted median | 8 | 1.04 | 0.95-1.14 | 0.356 |
| Simple median | 8 | 1.03 | 0.94-1.12 | 0.586 |
| MR-PRESSO test | 8 | 1.00 | 0.94-1.07 | 0.954 |
| MR-Egger | 8 | / | / | 0.388* |
| **MIF** |  |  |  |  |
| Inverse-variance weighted | 6 | 0.99 | 0.90-1.09 | 0.784 |
| Weighted median | 6 | 1.00 | 0.89-1.12 | 0.953 |
| Simple median | 6 | 0.99 | 0.88-1.12 | 0.891 |
| MR-PRESSO test | 6 | 0.99 | 0.91-1.07 | 0.765 |
| MR-Egger | 6 | / | / | 0.272* |
| **MIG** |  |  |  |  |
| Inverse-variance weighted | 17 | 0.96 | 0.91-1.01 | 0.092 |
| Weighted median | 17 | 0.92 | 0.86-0.99 | 0.025 |
| Simple median | 17 | 0.94 | 0.87-1.02 | 0.153 |
| MR-PRESSO test | 17 | 0.96 | 0.90-1.02 | 0.203 |
| MR-Egger | 17 | / | / | 0.002* |
| **MIP-1α** |  |  |  |  |
| Inverse-variance weighted | 11 | 1.03 | 0.96-1.11 | 0.421 |
| Weighted median | 11 | 1.01 | 0.91-1.12 | 0.856 |
| Simple median | 11 | 1.00 | 0.90-1.12 | 0.977 |
| MR-PRESSO test | 11 | 1.03 | 0.93-1.14 | 0.562 |
| MR-Egger | 11 | / | / | 0.500* |
| **MIP-1β** |  |  |  |  |
| Inverse-variance weighted | 70 | 1.01 | 0.97-1.04 | 0.664 |
| Weighted median | 70 | 0.98 | 0.93-1.04 | 0.564 |
| Simple median | 70 | 0.98 | 0.92-1.04 | 0.448 |
| MR-PRESSO test | 70 | 1.01 | 0.97-1.04 | 0.665 |
| MR-Egger | 70 | / | / | 0.233* |
| **PDGF-bb** |  |  |  |  |
| Inverse-variance weighted | 17 | 1.02 | 0.94-1.12 | 0.621 |
| Weighted median | 17 | 1.01 | 0.88-1.16 | 0.909 |
| Simple median | 17 | 1.00 | 0.86-1.15 | 0.949 |
| MR-PRESSO test | 17 | 1.02 | 0.91-1.15 | 0.702 |
| MR-Egger | 17 | / | / | 0.939* |
| **RANTES** |  |  |  |  |
| Inverse-variance weighted | 12 | 0.96 | 0.90-1.03 | 0.219 |
| Weighted median | 12 | 0.95 | 0.86-1.04 | 0.236 |
| Simple median | 12 | 0.95 | 0.87-1.04 | 0.289 |
| MR-PRESSO test | 12 | 0.96 | 0.90-1.02 | 0.215 |
| MR-Egger | 12 | / | / | 0.441* |
| **SCF** |  |  |  |  |
| Inverse-variance weighted | 9 | 0.96 | 0.86-1.07 | 0.462 |
| Weighted median | 9 | 0.99 | 0.85-1.15 | 0.884 |
| Simple median | 9 | 0.99 | 0.85-1.16 | 0.912 |
| MR-PRESSO test | 9 | 0.96 | 0.88-1.05 | 0.388 |
| MR-Egger | 9 | / | / | 0.789* |
| **SCGF-β** |  |  |  |  |
| Inverse-variance weighted | 21 | 0.99 | 0.94-1.04 | 0.646 |
| Weighted median | 21 | 0.98 | 0.91-1.05 | 0.566 |
| Simple median | 21 | 0.98 | 0.91-1.06 | 0.607 |
| MR-PRESSO test | 21 | 0.99 | 0.94-1.03 | 0.600 |
| MR-Egger | 21 | / | / | 0.825* |
| **SDF-1α** |  |  |  |  |
| Inverse-variance weighted | 9 | 1.04 | 0.93-1.16 | 0.501 |
| Weighted median | 9 | 1.06 | 0.93-1.21 | 0.391 |
| Simple median | 9 | 1.05 | 0.91-1.21 | 0.531 |
| MR-PRESSO test | 9 | 1.04 | 1.00-1.08 | 0.101 |
| MR-Egger | 9 | / | / | 0.744* |
| **TNF-α** |  |  |  |  |
| Inverse-variance weighted | 4 | 0.94 | 0.85-1.04 | 0.230 |
| Weighted median | 4 | 0.97 | 0.86-1.10 | 0.661 |
| Simple median | 4 | 0.98 | 0.86-1.10 | 0.703 |
| MR-PRESSO test | 4 | 0.94 | 0.85-1.04 | 0.306 |
| MR-Egger | 4 | / | / | 0.285* |
| **TNF-β** |  |  |  |  |
| Inverse-variance weighted | 6 | 0.97 | 0.91-1.03 | 0.304 |
| Weighted median | 6 | 0.97 | 0.90-1.04 | 0.357 |
| Simple median | 6 | 0.97 | 0.90-1.04 | 0.377 |
| MR-PRESSO test | 6 | 0.97 | 0.93-1.02 | 0.249 |
| MR-Egger | 6 | / | / | 0.183* |
| **TRAIL** |  |  |  |  |
| Inverse-variance weighted | 33 | 0.99 | 0.96-1.02 | 0.532 |
| Weighted median | 33 | 0.99 | 0.94-1.03 | 0.624 |
| Simple median | 33 | 0.98 | 0.93-1.04 | 0.593 |
| MR-PRESSO test | 33 | 0.99 | 0.96-1.02 | 0.499 |
| MR-Egger | 33 | / | / | 0.392* |
| **VEGF** |  |  |  |  |
| Inverse-variance weighted | 20 | 1.04 | 0.96-1.12 | 0.352 |
| Weighted median | 20 | 1.03 | 0.92-1.14 | 0.658 |
| Simple median | 20 | 1.09 | 0.97-1.22 | 0.160 |
| MR-PRESSO test | 20 | 1.04 | 0.96-1.12 | 0.356 |
| MR-Egger | 20 | / | / | 0.097* |

Abbreviations: β-NGF, beta nerve growth factor; CI, confidence interval; CTACK, cutaneous T-cell attracting (CCL27); FGF-basic, basic fibroblast growth factor; G-CSF, granulocyte colony-stimulating factor; GRO-a, growth regulated oncogene-α (CXCL1); HGF, hepatocyte growth factor; IFN-γ, interferon-gamma; IL-1rα, interleukin-1 receptor antagonist; IL-1β, interleukin-1-beta; IL-2, interleukin-2; IL-2rα, interleukin-2 receptor, alpha subunit; IL-4, interleukin-4; IL-5, interleukin-5; IL-6, interleukin-6; IL-7, interleukin-7; IL-8, interleukin-8; IL-9, interleukin-9; IL-10, interleukin-10; IL-12p70, interleukin-12p70; IL-13, interleukin-13; IL-16, interleukin-16; IL-17, interleukin-17; IL-18, interleukin-18; IP-10, interferon gamma-induced protein 10 (CXCL10); MCP-1, monocyte chemotactic protein-1; MCP-3, monocyte specific chemokine 3 (CCL7); M-CSF, macrophage colony-stimulating factor; MIF, macrophage migration inhibitory factor; MIG, monokine induced by interferon-gamma; MIP-1α, macrophage inflammatory protein-1α (CCL3); MIP-1b, macrophage inflammatory protein-1β; MR, Mendelian randomization; MR-PRESSO,MR pleiotropy residual sum and outlier; OR, odds ratio; PDGF-bb, platelet derived growth factor BB; RANTES, regulated on activation normal T Cell expressed and secreted (CCL5); SCF, stem cell factor; SCGF-β, stem cell growth factor beta; SDF-1α, stromal cell-derived factor-1 alpha; SNP, single nucleotide polymorphism; TNF-α, tumor necrosis factor-alpha; TNF-β, tumor necrosis factor-beta; TRAIL, TNF-related apoptosis inducing ligand; VEGF, vascular endothelial growth factor. **P*-value of the intercept from MR-Egger regression analysis.

**Table S6.** Details of the genetic variants with potential pleiotropy among instrumental variables of IL-2.

| Cytokines/ Growth factors | SNP | Pleiotropic trait^*^ | *P*-value | PMID |
| --- | --- | --- | --- | --- |
| IL-2 | rs1848347 | Hemoglobin | 6.00×10^-16^ | 29403010 |
|  |  | Red blood cell count | 1.00×10^-357^ | 32888493 |

^*^ From the GWAS Catalog (http://www.ebi.ac.uk/gwas, last accessed on June 27th, 2022).

**Table S7.** Effect estimates of the associations of circulating level of IL-2 with risk of amyotrophic lateral sclerosis after excluding potential pleiotropic SNPs.

| Methods | Number of SNPs | OR | 95% CI | *P-*value |
| --- | --- | --- | --- | --- |
| IL-2 |  |  |  |  |
| Inverse-variance weighted | 9 | 0.94 | 0.87-1.01 | 0.092 |
| Weighted median | 9 | 0.94 | 0.83-1.05 | 0.253 |
| Simple median | 9 | 0.94 | 0.84-1.06 | 0.326 |
| MR-PRESSO test | 9 | 0.94 | 0.85-1.04 | 0.241 |
| MR-Egger | 9 | \ | \ | 0.963* |

Abbreviations: CI, confidence interval; IL-2, interleukin-2; MR, Mendelian randomization; MR-PRESSO test, MR Pleiotropy RESidual Sum and Outlier test; OR, odds ratio; SNP, single nucleotide polymorphism. **P*-value of the intercept from MR-Egger regression analysis.

**Table S8.** Characteristics of the genetic variants associated with the amyotrophic lateral sclerosis.

| SNP | Chr | Position | Effect allele | Beta | SE | *P*-value |
| --- | --- | --- | --- | --- | --- | --- |
| rs10463311 | 5 | 150410835 | t | -0.0854 | 0.0156 | 4.00×10^-8^ |
| rs3849943 | 9 | 27543382 | t | -0.1764 | 0.0155 | 3.77×10^-30^ |
| rs118082508 | 12 | 57318819 | t | 0.3175 | 0.0565 | 1.97×10^-8^ |
| rs113247976 | 12 | 57975700 | t | 0.3221 | 0.0521 | 6.43×10^-10^ |
| rs142321490 | 12 | 58676132 | c | 0.3172 | 0.0513 | 6.15×10^-10^ |
| rs74654358 | 12 | 64881967 | a | 0.1976 | 0.0337 | 4.66×10^-9^ |
| rs12973192 | 19 | 17753239 | c | -0.1205 | 0.0153 | 3.92×10^-15^ |
| rs75087725 | 21 | 45753117 | a | 0.515 | 0.067 | 1.85×10^-14^ |

Abbreviations: Chr, chromosome; SE, standard error; SNP, single nucleotide polymorphism.

**Table S9.** Effect estimates of the associations of amyotrophic lateral sclerosis with risk of circulating levels of FGF-basic and IP-10.

| Methods | Number of SNPs | OR | 95% CI | *P-*value |
| --- | --- | --- | --- | --- |
| FGF-basic |  |  |  |  |
| Inverse-variance weighted | 8 | 0.87 | 0.79-0.97 | 0.015 |
| Weighted median | 8 | 0.87 | 0.76-1.00 | 0.050 |
| Simple median | 8 | 0.86 | 0.75-0.99 | 0.032 |
| MR-PRESSO test | 8 | 0.88 | 0.82-0.93 | 0.003 |
| MR-Egger | 8 | \ | \ | 0.822* |
| IP-10 |  |  |  |  |
| Inverse-variance weighted | 8 | 0.92 | 0.79-1.08 | 0.302 |
| Weighted median | 8 | 0.92 | 0.74-1.14 | 0.437 |
| Simple median | 8 | 0.86 | 0.70-1.06 | 0.159 |
| MR-PRESSO test | 8 | 0.92 | 0.76-1.11 | 0.427 |
| MR-Egger | 8 | \ | \ | 0.234* |

Abbreviations: CI, confidence interval; FGF-basic, basic fibroblast growth factor; IP-10, interferon gamma-induced protein 10 (CXCL10); MR-PRESSO test, MR Pleiotropy RESidual Sum and Outlier test; OR, odds ratio; SNP, single nucleotide polymorphism. **P*-value of the intercept from MR-Egger regression analysis.
